# The Effect of Total Cholesterol Variability on Clinical Outcomes After Percutaneous Coronary Intervention

**DOI:** 10.3389/fpubh.2022.804031

**Published:** 2022-02-08

**Authors:** Yanting Liang, Haochen Wang, Fengyao Liu, Xueju Yu, Yan Liang, Han Yin, Yuting Liu, Cheng Jiang, Yu Wang, Bingqing Bai, Anbang Liu, Xiaohe Shi, Weiya Li, Quanjun Liu, Yilin Chen, Lan Guo, Huan Ma, Qingshan Geng

**Affiliations:** ^1^Department of Cardiology, Guangdong Cardiovascular Institute, Guangdong Provincial People's Hospital, Guangdong Academy of Medical Sciences, Guangzhou, China; ^2^Department of Geriatrics, Guangdong Provincial Geriatrics Institute, Guangdong Provincial People's Hospital, Guangdong Academy of Medical Sciences, Guangzhou, China; ^3^School of Medicine, South China University of Technology, Guangzhou, China

**Keywords:** total cholesterol variability, coronary artery disease, percutaneous coronary intervention, clinical outcomes, major adverse cardiovascular, cerebrovascular events

## Abstract

**Aim:**

Exploring the risk factors of prognosis in patients undergoing percutaneous coronary intervention (PCI) is of great importance. Our aim of the study is to investigate the association between variability in total cholesterol (TC) level and major adverse cardiovascular and cerebrovascular events (MACCE) in patients after PCI.

**Methods:**

Between April 2004 and December 2009, 909 patients who underwent primary PCI and with at least three TC values were included in the final study. TC variability was calculated using four indices: standard deviation (SD), coefficient of variation (CV), the average successive variability (ASV), variability independent of the mean (VIM). MACCE comprised all-cause mortality, non-fatal myocardial infarction (MI), unplanned revascularization, hospitalization for heart failure, and non-fatal stroke.

**Results:**

There were 394 cases of MACCE during the follow-up period. When the subjects were divided into quartile groups by CV of TC, high CV groups were associated with a higher hazard ratio of MACCE than for lower CV groups. In multivariable adjusted models, TC variability and MACCE remained correlated [HR (95% CI): Q2, 1.17 (0.86–1.58); Q3, 1.38 (1.03–1.85); Q4, 1.63 (1.22–2.17)]. Similar patterns of MACCE were noted by quartiles of SD, ASV, and VIM.

**Conclusion:**

Visit-to-visit TC variability is positively correlated with MACCE in patients after PCI.

## Introduction

Percutaneous coronary intervention (PCI) is the major therapy of coronary artery disease (CAD) (1). However, there are still a significant proportion of patients with major adverse cardiovascular and cerebrovascular events (MACCE) after PCI (2). In order to reduce the incidence of MACCE, it is particularly important to explore the risk factors of prognosis. The main risk factors include diabetes mellitus (DM), hypertension, dyslipidaemia, smoking, obesity, and so on (3–7).

Abnormal blood lipid metabolism is a risk factor for CAD (8). It has been well-established that high concentrations of total cholesterol (TC) are associated with greater CAD risk. Serum total cholesterol contribute to atherosclerosis and fluctuations of blood cholesterol level may be independently associated with adverse outcomes (9, 10). Therefore, monitoring and management blood lipid after PCI is essential.

Recently, the visit-to-visit cholesterol variability is under the spotlight. A high visit-to-visit variability in cholesterol levels was suggested to be an independent predictor of major adverse cardiovascular events (MACE) (11). A previous study showed that the visit-to-visit variability in fasting measurements of high-density lipoprotein cholesterol (HDL-C), triglycerides (TG), and low-density lipoprotein cholesterol (LDL-C) are predictive of coronary events (12). A study demonstrated that, for general population, total cholesterol (TC) variability is a vital risk factor (11). Another study suggested that the visit-to-visit variability in LDL-C, HDL-C, and non-HDL-C in post-PCI patients can be considered as a predictor of adverse cardiovascular events (13). However, few previous studies have directly assessed the role of TC variability as a predictor of MACCE among post-PCI patients. Therefore, we sought to evaluate the prognostic impact of TC variability in patients undergoing PCI.

## Methods

Research was carried out according to The Code of Ethics of the World Medical Association (Declaration of Helsinki) and was approved by the Guangdong Provincial People's Hospital Ethics Committee. Written consents were obtained from participating patients.

### Patients

In this study, we recruited patients from “CHD club” of Guangdong Cardiovascular Institute, Guangdong Provincial People's Hospital. Subjects were those CHD patients who have had PCI between April 2004 and December 2009. CHD secondary prevention educations were provided to patients by a regular doctor in the “CHD club,” which include lifestyle adjustment instructions and drug treatments. Biochemical investigations were also conducted during hospitalization and 1, 3, 6, 12, 24, and 36 months after PCI. All blood lipid measurements were carried out in the same testing institution to avoid bias caused by different measuring instruments. Among the 2,258 patients enrolled in this club, 909 patients with at least three TC values were included in the final study. Baseline data was collected from the time of PCI.

### PCI and Medications

PCI was performed by experienced interventional cardiologists according to standard national practice as described in previous researches (14). The use of statins, antiplatelet agents (aspirin/clopidogrel), β-adrenergic blocking agents, angiotensin-converting enzyme inhibitors, diuretics, or inotropic drugs was based on the discretion of the attending cardiologist according to clinical protocol derived from national interventional guidelines.

### Variability Measurements

TC values were measured during hospitalization and 1, 3, 6, 12, 24, and 36 months after PCI. In this study, patients completing the baseline TC values and at least 2 post-baseline TC values were included in analyses. TC variability was defined as intra-individual variability in TC values between visits. Four indices of variability were used: (1) standard deviation (SD), (2) coefficient of variation (CV), (3) the average successive variability (ASV), which refers to the average absolute difference between successive values, (4) variability independent of the mean (VIM). VIM was calculated as 100 × SD/Mean^beta^, where beta is the regression coefficient, on the basis of natural logarithm of SD on the natural logarithm of mean (15). The mean of TC measurements is 4.64 and the number of TC measurements per patient ranged from 3 to 7: 3 measurements (*n* = 247, 27.2%), 4 measurements (*n* = 221, 24.3%), 5 measurements (*n* = 177, 19.5%), and 6 measurements (*n* = 139, 15.3%), 6 measurements (*n* = 139, 14.3%), and 7 measurements (*n* = 125, 13.8%).

### Clinical Outcomes and Follow-Up

Annual telephone follow-up was conducted by trained research assistants for all participants according to standardized protocols, which guaranteed the reliability of the survey. The endpoint of the study was the major adverse cardiovascular and cerebrovascular events (MACCE). Components of MACCE included all-cause mortality, myocardial infarction (MI), unplanned revascularization, hospitalization for heart failure, and non-fatal stroke.

### Statistical Analysis

We categorized subjects into four groups according to the quartile distribution of TC variability. The Kaplan-Meier curves was used to analyze survival and disease-free probability, and the log-rank test was performed to analyze the significance of the difference between groups. The Cox proportional hazards regression model was performed to calculate the 95% confidence interval (95% Cl) and hazard ratios (HR) values of different outcomes. Three multivariable models were conducted: in Model one, age, sex, BMI, and smoking history were adjusted; Model two was adjusted for the variables in model one plus diabetes and hypertension; Model three was adjusted for the variables in model two plus mean TC value and lipid-lowering agents. Sensitivity analyses were also performed. Statistical analyses were performed using SPSS (IBM SPSS 26.0, SPSS Inc). A *P* < 0.05 was considered statistically significant.

## Results

### Baseline Characteristics of Participants

Follow-up ranged from 48 to 59 months (median follow-up, 53 months). Table 1 shows characteristics of subjects according to CV quartiles. Subjects with higher TC variability were more likely to have dyslipidaemia and need to use lipid-lowering agents more frequently. These patients were more frequently atorvastatin treatment. There were no statistically significant differences of mean TC level between groups (4.5 ± 0.9 mmol/L). The CV values of TC in the Q1-Q4 groups were 6.1 ± 2.4, 12 ± 1.3, 16.8 ± 1.7, and 25.9 ± 5.5%, respectively. We found that there is no significant difference in the first TC measurement months after PCI between quartiles. The first TC measurement of the included patients usually measured in 1 months after PCI (Supplementary Table S6). For details of baseline characteristics according to quartiles of SD, ASV and VIM, see Supplementary Materials (Supplementary Tables S1–3).

**Table 1:** Baseline characteristics of subjects to the total cholesterol variability (coefficient of variation).

	**Q 1 (*n =* 228)**	**Q 2 (*n =* 227)**	**Q 3 (*n =* 227)**	**Q 4 (*n =* 227)**	**Overall (*n =* 909)**	***P*-value**
Age (years)	64.2 ± 10	64.2 ± 10.4	63.6 ± 9.2	61.9 ± 10.9	63.5 ± 10.2	0.077
Sex (male)	164 (71.9)	178 (78.4)	179 (78.9)	171 (75.3)	692 (76.1)	0.276
Body mass index (kg/m^2^)	23.9 ± 2.9	23.9 ± 2.9	24 ± 3.2	24.2 ± 3	24 ± 3	0.598
Mean TC (mmol/L)	4.4 ± 0.8	4.4 ± 0.9	4.5 ± 0.8	4.5 ± 0.9	4.5 ± 0.9	0.617
**TC variability**						
CV (%)	6.1 ± 2.4	12 ± 1.3	16.8 ± 1.7	25.9 ± 5.5	15.2 ± 7.9	<0.001
SD (mmol/L)	0.3 ± 0.1	0.5 ± 0.1	0.8 ± 0.2	1.2 ± 0.4	0.7 ± 0.4	<0.001
ASV (mmol/L)	0.3 ± 0.2	0.6 ± 0.2	0.8 ± 0.3	1.3 ± 0.6	0.7 ± 0.5	<0.001
VIM (%)	4.5 ± 1.8	9 ± 1	12.5 ± 1.3	19.2 ± 4.1	11.3 ± 5.9	<0.001
Current smoker	71 (31.1)	75 (33)	84 (37)	80 (35.2)	310 (34.1)	0.575
Hypertension	200 (87.7)	208 (91.6)	198 (87.2)	195 (85.9)	801 (88.1)	0.267
Diabetes mellitus	51 (22.4)	50 (22)	62 (27.3)	64 (28.2)	227 (25)	0.284
Dyslipidaemia	44 (19.3)	59 (26)	79 (34.8)	106 (46.7)	288 (31.7)	<0.001
On lipid-lowering agent	160 (70.2)	190 (83.7)	187 (82.4)	180 (79.3)	717 (78.9)	0.002
**Lipid-lowering agent type**						0.245
Atorvastatin	73 (45.3)	99 (52.4)	101 (54.3)	105 (58)	378 (52.7)	–
Simvastatin	57 (35.4)	64 (33.9)	49 (26.3)	54 (29.8)	224 (31.2)	–
Pravastatin	11 (6.8)	14 (7.4)	12 (6.5)	7 (3.9)	44 (6.1)	–
Fluvastatin	15 (9.3)	6 (3.2)	15 (8.1)	10 (5.5)	46 (6.4)	–
Other	5 (3.1)	6 (3.2)	9 (4.8)	5 (2.8)	25 (3.5)	–
The first TC measurement months after PCI	1.9 ± 1.2	1.7 ± 1.0	1.8 ± 1.1	1.9 ± 1.1	1.8 ± 1.1	0.212

*Data are expressed as the mean ± SD, or n (%)*.

*TC, total cholesterol; CV, coefficient of variation; SD, standard deviation; ASV, the average successive variability; VIM, variability independent of the mean*.

### Visit-to-Visit TC Variability and MACCE

**Figure 1:**
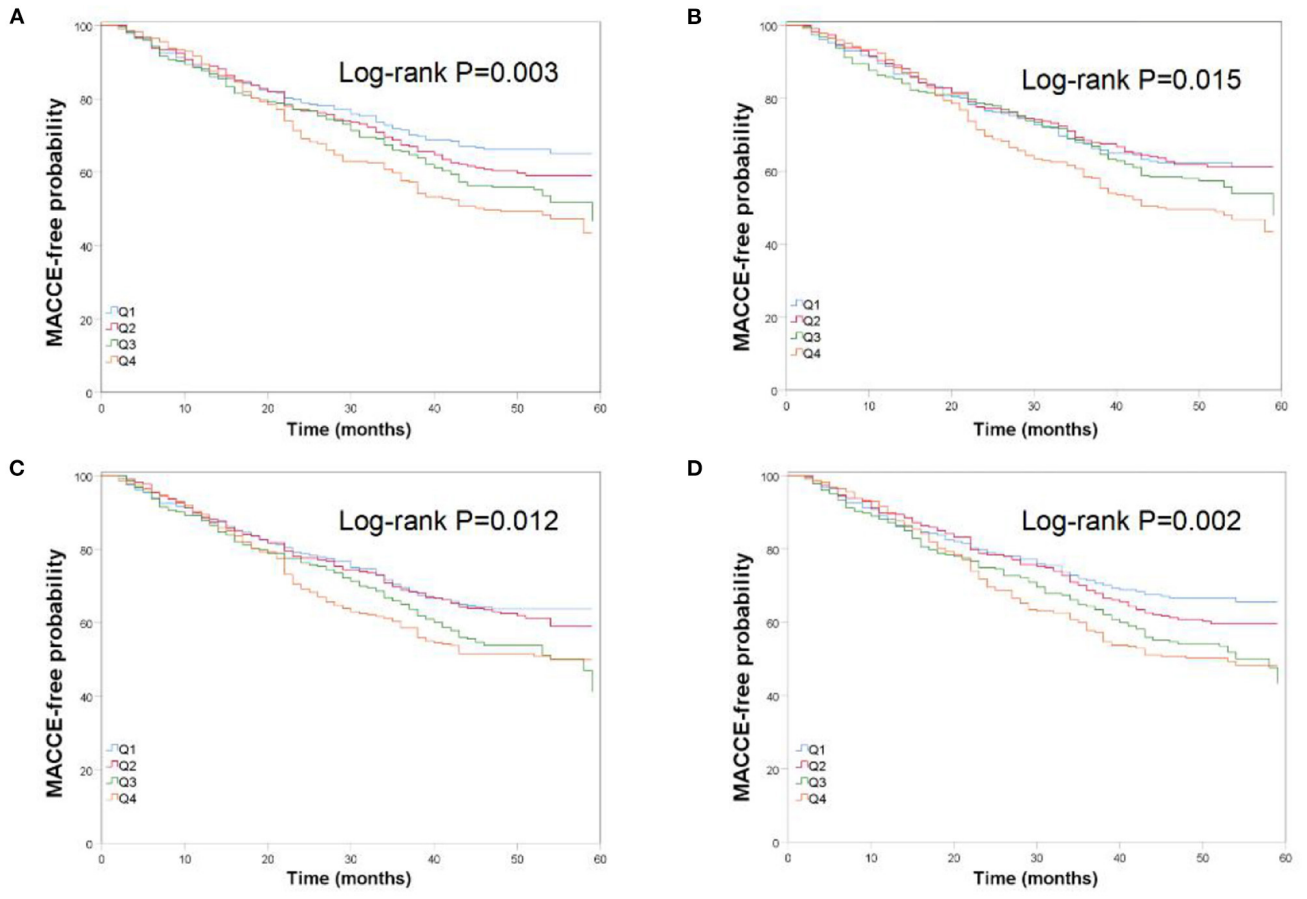
Kaplan-Meier estimates of MACCE-free probability by quartiles of total cholesterol variability measured as four indices: **(A)** K-M curve by CV; **(B)** K-M curve by SD; **(C)** K-M curve by ASV; **(D)** K-M curve by VIM.

There were 394 cases of MACCE during the follow-up period. When the subjects were divided into quartile groups by CV of TC, high CV groups were associated with a higher hazard ratio of MACCE than the lower CV groups. TC variability and MACCE still remained correlated in Model 4 [HR (95% CI): Q2, 1.17 (0.86–1.58); Q3, 1.38 (1.03–1.85); Q4, 1.63 (1.22–2.17)]. Similar patterns of MACCE were noted by quartiles of SD, ASV and VIM. An additive effect of the variability of TC on the risk of MACCE was identified (Table 2). The Kaplan-Meier cumulative MACCE-free survival curves by quartiles of TC variability measured as different indices showed that the presence of high variability of TC was associated with higher incidences of MACCE after PCI (Figure 1).

**Table 2:** Hazard ratios and 95% confidence intervals of MACCE by quartiles of total cholesterol variability.

	**Events**	**Crude**		**Model 1**		**Model 2**		**Model 3**	
		**HR (95% CI)**	***P*-value**	**HR (95% CI)**	***P*-value**	**HR (95% CI)**	***P*-value**	**HR (95% CI)**	***P*-value**
**CV**									
Q 1	78	1 (ref)	0.003	1 (ref)	0.003	1 (ref)	0.003	1 (ref)	0.005
Q 2	92	1.22 (0.90–1.64)	0.204	1.20 (0.89–1.62)	0.242	1.20 (0.89–1.62)	0.242	1.17 (0.86–1.58)	0.316
Q 3	106	1.43 (1.07–1.91)	0.017	1.40 (1.05–1.88)	0.024	1.40 (1.05–1.88)	0.024	1.38 (1.03–1.85)	0.034
Q 4	118	1.68 (1.26–2.23)	<0.001	1.67 (1.25–2.22)	<0.001	1.67 (1.25–2.22)	<0.001	1.63 (1.22–2.17)	0.001
P for trend			0.003		0.001	0.001	0.001	0.001	0.001
**SD**									
Q 1	88	1 (ref)	0.019	1 (ref)	0.012	1 (ref)	0.012	1 (ref)	0.015
Q 2	88	1.01 (0.75–1.35)	0.97	1 (0.74–1.34)	0.98	1 (0.74–1.34)	0.98	0.98 (0.73–1.31)	0.875
Q 3	101	1.19 (0.89–1.58)	0.243	1.18 (0.89–1.57)	0.256	1.18 (0.89–1.57)	0.256	1.15 (0.86–1.53)	0.354
Q 4	117	1.47 (1.11–1.94)	0.006	1.49 (1.13–1.97)	0.005	1.49 (1.13–1.97)	0.005	1.46 (1.11–1.93)	0.007
P for trend			0.019		0.004		0.004		0.001
**ASV**									
Q 1	87	1 (ref)	0.012	1 (ref)	0.008	1 (ref)	0.008	1 (ref)	0.015
Q 2	86	1.09 (0.81–1.46)	0.59	1.09 (0.81–1.47)	0.559	1.09 (0.81–1.47)	0.559	1.07 (0.79–1.44)	0.659
Q 3	109	1.41 (1.06–1.87)	0.017	1.4 (1.06–1.86)	0.019	1.4 (1.06–1.86)	0.019	1.36 (1.03–1.81)	0.031
Q 4	112	1.48 (1.12–1.96)	0.006	1.53 (1.16–2.03)	0.003	1.53 (1.16–2.03)	0.003	1.49 (1.12–1.97)	0.006
P for trend			0.012		0.002		0.002		0.001
**VIM**									
Q 1	77	1 (ref)	0.002	1 (ref)	0.002	1 (ref)	0.002	1 (ref)	0.004
Q 2	91	1.2 (0.89–1.63)	0.234	1.2 (0.89–1.63)	0.234	1.2 (0.89–1.63)	0.234	1.16 (0.86–1.57)	0.34
Q 3	111	1.54 (1.15–2.06)	0.004	1.54 (1.15–2.06)	0.004	1.54 (1.15–2.06)	0.004	1.49 (1.11–2)	0.007
Q 4	115	1.65 (1.24–2.21)	0.001	1.65 (1.24–2.21)	0.001	1.65 (1.24–2.21)	0.001	1.6 (1.19–2.13)	0.002
P for trend			0.002		0.002		0.002		<0.001

*Model 1: adjusted for age, sex, body mass index and smoking history*.

*Model 2: adjusted for model 1 plus diabetes mellitus and hypertension*.

*Model 3: adjusted for model 2 plus mean total cholesterol level and the use of lipid-lowering agents*.

*MACCE, the major adverse cardiovascular and cerebrovascular events; HR, hazard ratio; CI, confidence intervals; CV, coefficient of variation; SD, standard deviation; ASV, the average successive variability; VIM, variability independent of the mean*.

### Sensitivity Analysis

Findings were similar when SD, ASV, and VIM were used to determine the variability of TC. Even after full multivariable adjustment, TC variability as measured by SD, ASV, or VIM was also an independent predictor of MACCE (Table 2, Figure 1). The relation between TC variability and MACCE still remained significant when excluding subjects with dyslipidaemia (see Supplementary Material, Supplementary Table S4). Furthermore, we performed time-dependent Cox regression analysis. Similar results were observed when using the mean TC value as time-varying covariates (see Supplementary Materials online, Supplementary Table S5).

## Discussion

To our knowledge, this is the first study reporting the relationship between visit-to-visit TC variability and long-term cardiovascular outcomes in patients with previous PCI. In this study, we demonstrated that TC variability was significantly associated with increased risk for MACCE in patients with previous PCI, even after adjustment for age, sex, BMI, smoking history, diabetes, hypertension, and lipid-lowering agents. The results remained significant when use other indices of TC variability and by several sensitivity analyses. The relationship between TC variability and MACCE still remained significant when subjects with dyslipidaemia were excluded. Therefore, it is important to monitoring variability in cholesterol levels among post-PCI patients without dyslipidaemia. This finding indicates that visit-to-visit TC variability may be novel biometrics for monitoring response to lipid-lowering therapy and a predictor of MACCE in post-PCI patients.

Acute coronary syndrome (ACS) is one of leading cause of death in the world (16). PCI has tremendous progress on the management of patients with ACS, which dramatically reduces the cardiovascular mortality and disability rates (17, 18). However, there are still life-threatening complications after PCI, such as death, MI and stroke (19). Therefore, it is necessary to identify modifiable risk factors for prognosis in post-PCI patients (20–22).

Variability in biological parameters has been considered as biomarkers with the prognostic value in many studies. Although these parameters are different in mechanisms, these fluctuations in biological indicators such as blood pressure, heart rate, and lipid levels may contribute to and be predictors of clinical outcomes. Visit-to-visit blood pressure variability is studied the most (23–25). In a randomized controlled trial of 13,803 patients with hypertension, the level of visit-to-visit systolic blood pressure variability was reported to be associated with the risk of cardiovascular events (24). Although cholesterol level is considered one of the most contributing risk factors for the prognosis of PCI, the correlation between cholesterol variability and CVD has been little investigated, especially in patients who underwent PCI.

A study with 3,656,648 subjects reported that in the multivariable adjusted model, the HR and 95% CI of CV for all-cause mortality, MI, and stroke were, respectively, 1.26 (1.24–1.28), 1.08 (1.05–1.11), and 1.11 (1.08–1.14) (11). In our study, results shows that in post-PCI patients, the HR and 95% CI for the highest vs. lowest quartiles of CV of TC are 1.63 (1.22–2.17) for MACCE. It indicates that TC variability level may have a greater impact on adverse clinical outcomes in patients after PCI than in the general population. In another study with 1,792 subjects, LDL-, HDL-, and non-HDL-cholesterol variability was demonstrated to be significantly associated with increased risk for MACCE in post-PCI patients (13). Our study finds that the level of TC variability is also an independent predictor of MACCEs in patients who underwent PCI. Further study is required to determine which cholesterol variability is the best.

### Limitations

The retrospective single-center design is the main limitation of our study. To overcome this limitation, more rigorous and long-term multicenter studies are needed in the future. Secondly, dosage information of lipid-lowering agents was unavailable. Thirdly, the information about dietary habits and medication compliance was not available. These limitations make it hard for us to analyze the factors contributing to high variability. Lastly, only the Chinese population was included in the study and therefore our results cannot be extrapolated to other ethnicities.

## Conclusions

In conclusion, TC variability is a predictor of MACCE in patients who underwent PCI. The results of this study provide new clinical significance for continuous monitoring of TC levels in patients after PCI. Future studies should explore factors influencing lipid variability and clarify the relationship between interventions to reduce lipid variability and clinical outcomes. The development of long-acting lipid-lowering drugs may be of great help.

## Data Availability Statement

The raw data supporting the conclusions of this article will be made available by the authors, without undue reservation.

## Ethics Statement

The studies involving human participants were reviewed and approved by Guangdong Provincial People's Hospital Ethics Committee. The patients/participants provided their written informed consent to participate in this study. Written informed consent was obtained from the individual(s) for the publication of any potentially identifiable images or data included in this article.

## Author Contributions

YantL, HW, and FL performed and interpreted statistical analysis and drafted manuscript writing. YantL, FL, HW, XY, YanL, HY, YuL, CJ, YW, BB, AL, XS, WL, and YC collected and entered data into database. LG, HM, and QG were senior physicians principally responsible for the study. HM and QG revised the paper. All authors read and approved the final manuscript.

## Funding

This work was supported by the grants of the National Natural Science Foundation of China (Grant No. 8160284), Natural Science Foundation of Guangdong Province (Grant No. 2019A15 15011224), and Traditional Chinese Medicine Bureau of Guangdong Province (Grant 20201008).

## Conflict of Interest

The authors declare that the research was conducted in the absence of any commercial or financial relationships that could be construed as a potential conflict of interest.

## Publisher's Note

All claims expressed in this article are solely those of the authors and do not necessarily represent those of their affiliated organizations, or those of the publisher, the editors and the reviewers. Any product that may be evaluated in this article, or claim that may be made by its manufacturer, is not guaranteed or endorsed by the publisher.
