# Supplementary material for: The Effect of Total Cholesterol Variability on Clinical Outcomes After Percutaneous Coronary Intervention
Source: Front Public Health. 2022 Feb 8;10:804031. doi: 10.3389/fpubh.2022.804031 (PMC8860968; doi:10.3389/fpubh.2022.804031)
Supplement: Supplementary file 1 [file Data_Sheet_1.PDF]

Supplementary material

Supplementary Table 1 Baseline characteristics of subjects to the total cholesterol variability (standard deviation)

	Q 1 (n = 231)	Q 2 (n = 228)	Q 3 (n = 226)	Q 4 (n = 224)	Overall (n = 909)	P value
Age (years)	64.8±9.9	63.7±10.6	64±9.2	61.4±10.7	63.5±10.2	0.005
Sex (male)	175(75.8)	182(79.8)	174(77)	161(71.9)	692(76.1)	0.256
Body mass index (kg/m^2)	23.8±2.8	24±3.1	24±3	24.2±3.1	24±3	0.532
Mean TC (mmol/L)	4.2±0.8	4.3±0.8	4.5±0.8	4.9±0.9	4.5±0.9	<0.001
**TC variability**						
CV (%)	6.4±2.9	12.4±2.5	17±3.1	25.2±6.2	15.2±7.9	<0.001
SD (mmol/L)	0.3±0.1	0.5±0.1	0.8±0.1	1.2±0.3	0.7±0.4	<0.001
ASV (mmol/L)	0.3±0.1	0.3±0.1	0.3±0.1	0.3±0.1	0.3±0.1	<0.001
VIM (%)	4.9±2.3	9.3±2.2	12.7±2.7	18.5±4.8	11.3±5.9	<0.001
Current smoker	67(29)	85(37.3)	84(37.2)	74(33)	310(34.1)	0.191
Hypertension	206(89.2)	203(89)	204(90.3)	188(83.9)	801(88.1)	0.159
Diabetes mellitus	56(24.2)	46(20.2)	62(27.4)	63(28.1)	227(25)	0.188
Dyslipidaemia	30(13)	52(22.8)	87(38.5)	119(53.1)	288(31.7)	<0.001
On lipid-lowering agent	170(73.6)	182(79.8)	187(82.7)	178(79.5)	717(78.9)	0.108

Data are expressed as the mean ± SD, or n (%).

TC, total cholesterol; CV, coefficient of variation; SD, standard deviation; ASV, the average successive variability; VIM, variability independent of the mean.

Supplementary Table 2 Baseline characteristics of subjects to the total cholesterol variability (the average successive variability)

	Q 1 (n = 240)	Q 2 (n = 219)	Q 3 (n = 223)	Q 4 (n = 227)	Overall (n = 909)	P value
Age (years)	64.6±10	63.4±10.3	63.8±9.8	62±10.5	63.5±10.2	0.039
Sex (male)	188(78.3)	169(77.2)	180(80.7)	155(68.3)	692(76.1)	0.011
Body mass index (kg/m^2)	23.9±2.9	24.1±2.9	23.9±3.2	24.2±3.1	24±3	0.601
Mean TC (mmol/L)	4.1±0.8	4.3±0.7	4.5±0.8	5±0.9	4.5±0.9	<0.001
**TC variability**						
CV (%)	7.3±3.9	13.3±4.2	16.9±4.8	23.7±7.1	15.2±7.9	<0.001
SD (mmol/L)	0.3±0.1	0.6±0.2	0.7±0.2	1.2±0.4	0.7±0.4	<0.001
ASV (mmol/L)	0.3±0.1	0.5±0.1	0.8±0.1	1.4±0.5	0.7±0.5	<0.001
VIM (%)	5.5±3.1	10±3.3	12.6±3.9	17.3±5.4	11.3±5.9	<0.001
Current smoker	72(30)	78(35.6)	84(37.7)	76(33.5)	310(34.1)	0.345
Hypertension	212(88.3)	196(89.5)	204(91.5)	189(83.3)	801(88.1)	0.047
Diabetes mellitus	53(22.1)	49(22.4)	66(29.6)	59(26)	227(25)	0.21
Dyslipidaemia	34(14.2)	51(23.3)	78(35)	125(55.1)	288(31.7)	<0.001
On lipid-lowering agent	176(73.3)	173(79)	182(81.6)	186(81.9)	717(78.9)	0.082

Data are expressed as the mean ± SD, or n (%).

TC, total cholesterol; CV, coefficient of variation; SD, standard deviation; ASV, the average successive variability; VIM, variability independent of the mean.

Supplementary Table 3 Baseline characteristics of subjects to the total cholesterol variability (variability independent of the mean)

	Q 1 (n = 228)	Q 2 (n = 227)	Q 3 (n = 227)	Q 4 (n = 227)	Overall (n = 909)	P value
Age (years)	64.1±10.2	63.8±10.1	63.9±9.3	61.9±10.9	63.5±10.2	0.089
Sex (male)	164(71.9)	174(76.7)	181(79.7)	173(76.2)	692(76.1)	0.276
Body mass index (kg/m^2)	23.9±2.8	23.9±3	24.1±3.2	24.2±3	24±3	0.638
Mean TC (mmol/L)	4.5±0.9	4.5±0.9	4.5±0.8	4.4±0.9	4.5±0.9	0.877
**TC variability**						
CV (%)	6.1±2.5	12.1±1.4	16.8±1.8	25.8±5.5	15.2±7.9	<0.001
SD (mmol/L)	0.3±0.1	0.5±0.1	0.8±0.2	1.2±0.4	0.7±0.4	<0.001
ASV (mmol/L)	0.3±0.2	0.6±0.2	0.8±0.3	1.2±0.6	0.7±0.5	<0.001
VIM (%)	4.5±1.8	8.9±1	12.5±1.2	19.2±4.1	11.3±5.9	<0.001
Current smoker	72(31.6)	72(31.7)	81(35.7)	85(37.4)	310(34.1)	0.457
Hypertension	200(87.7)	207(91.2)	198(87.2)	196(86.3)	801(88.1)	0.402
Diabetes mellitus	51(22.4)	53(23.3)	60(26.4)	63(27.8)	227(25)	0.505
Dyslipidaemia	46(20.2)	62(27.3)	80(35.2)	100(44.1)	288(31.7)	<0.001
On lipid-lowering agent	160(70.2)	187(82.4)	186(81.9)	184(81.1)	717(78.9)	0.003

Data are expressed as the mean ± SD, or n (%).

TC, total cholesterol; CV, coefficient of variation; SD, standard deviation; ASV, the average successive variability; VIM, variability independent of the mean.

Supplementary Table 4 Hazard ratios and 95% confidence intervals of MACCE by quartiles of total cholesterol variability: Sensitivity analysis excluding all subjects with dyslipidaemia

	Events	Crude		Model 1		Model 2		Model 3	
		HR (95% CI)	P value	HR (95% CI)	P value	HR (95% CI)	P value	HR (95% CI)	P value
**CV**									
Q1	50		0.004		0.004		0.004		0.004
Q2	64	1.35 (0.93-1.95)	0.115	1.35 (0.93-1.95)	0.115	1.35 (0.93-1.95)	0.115	1.35 (0.93-1.95)	0.115
Q3	71	1.52 (1.06-2.18)	0.024	1.52 (1.06-2.18)	0.024	1.52 (1.06-2.18)	0.024	1.52 (1.06-2.18)	0.024
Q4	83	1.89 (1.33-2.68)	< 0.001	1.89 (1.33-2.68)	< 0.001	1.89 (1.33-2.68)	< 0.001	1.89 (1.33-2.68)	< 0.001
P for trend			0.004		0.004		0.004		0.004
**SD**									
Q1	54		0.001		0.001		0.001		0.001
Q2	68	1.4 (0.98-2)	0.065	1.4 (0.98-2)	0.065	1.4 (0.98-2)	0.065	1.4 (0.98-2)	0.065
Q3	59	1.18 (0.81-1.7)	0.385	1.18 (0.81-1.7)	0.385	1.18 (0.81-1.7)	0.385	1.18 (0.81-1.7)	0.385
Q4	87	1.92 (1.37-2.69)	< 0.001	1.92 (1.37-2.69)	< 0.001	1.92 (1.37-2.69)	< 0.001	1.92 (1.37-2.69)	< 0.001
P for trend			0.001		0.001		0.001		0.001
**ASV**									
Q1	52		0.004		0.004		0.004		0.004
Q2	67	1.47 (1.03-2.12)	0.036	1.47 (1.03-2.12)	0.036	1.47 (1.03-2.12)	0.036	1.47 (1.03-2.12)	0.036
Q3	67	1.38 (0.96-1.98)	0.081	1.38 (0.96-1.98)	0.081	1.38 (0.96-1.98)	0.081	1.38 (0.96-1.98)	0.081
Q4	82	1.87 (1.32-2.65)	< 0.001	1.87 (1.32-2.65)	< 0.001	1.87 (1.32-2.65)	< 0.001	1.87 (1.32-2.65)	< 0.001
P for trend			0.004		0.004		0.004		0.004
**VIM**									
Q1	50		0.004		0.004		0.004		0.004
Q2	63	1.31 (0.91-1.9)	0.15	1.31 (0.91-1.9)	0.15	1.31 (0.91-1.9)	0.15	1.31 (0.91-1.9)	0.15
Q3	74	1.59 (1.11-2.28)	0.011	1.59 (1.11-2.28)	0.011	1.59 (1.11-2.28)	0.011	1.59 (1.11-2.28)	0.011
Q4	81	1.85 (1.3-2.63)	0.001	1.85 (1.3-2.63)	0.001	1.85 (1.3-2.63)	0.001	1.85 (1.3-2.63)	0.001
P for trend			0.004		0.004		0.004		0.004

Model 1: adjusted for age, sex, body mass index and smoking

Model 2: adjusted for model 1 plus diabetes mellitus and hypertension

Model 3: adjusted for model 2 plus mean total cholesterol level and the use of lipid-lowering agents

MACCE, the major adverse cardiovascular and cerebrovascular events; HR, hazard ratio; CI, confidence intervals; CV, coefficient of variation; SD, standard deviation; ASV, the average successive variability; VIM, variability independent of the mean.

Supplementary Table 5 Hazard ratios and 95% confidence intervals of MACCE by quartiles of total cholesterol variability: Time-dependent Cox regression analysis

	CV		SD		ASV		VIM	
	HR (95%CI)	P value	HR (95%CI)	P value	HR (95%CI)	P value	HR (95%CI)	P value
Q1		0.003		0.001		0.001		0.002
Q2	1.22 (0.9 - 1.65)	0.191	1.04 (0.78 - 1.4)	0.78	1.14 (0.84-1.54)	0.4	1.21 (0.89 - 1.64)	0.222
Q3	1.44 (1.07 - 1.93)	0.015	1.27 (0.95-1.7)	0.104	1.5 (1.13-2)	0.005	1.54 (1.15 - 2.06)	0.004
Q4	1.69 (1.27 - 2.25)	< 0.001	1.7 (1.27-2.28)	< 0.001	1.74 (1.29-2.34)	< 0.001	1.64 (1.23 - 2.19)	0.001
P for trend		0.003		0.002		0.001		0.003

Adjusted for age, sex, body mass index, smoking, diabetes mellitus, hypertension, mean total cholesterol level and the use of lipid-lowering agents.

MACCE, the major adverse cardiovascular and cerebrovascular events; HR, hazard ratio; CI, confidence intervals; CV, coefficient of variation; SD, standard deviation; ASV, the average successive variability; VIM, variability independent of the mean.

Supplementary Table 6 Distribution of the first TC measurement months after PCI in the total cholesterol variability (coefficient of variation)

	Q 1 (n = 228)	Q 2 (n = 227)	Q 3 (n = 227)	Q 4 (n = 227)	Overall (n = 909)
**The first TC measurement months after PCI**					
1	127 (55.7)	137 (60.4)	128 (56.4)	120 (52.9)	512 (56.3)
2	43 (18.9)	47 (20.7)	49 (21.6)	53 (23.3)	192 (21.1)
3	33 (14.5)	30 (13.2)	29 (12.8)	31 (13.7)	123 (13.5)
4	18 (7.9)	10 (4.4)	16 (7.0)	14 (6.2)	58 (6.4)
5	5 (2.2)	2 (0.9)	3 (1.3)	9 (4.0)	9 (2.1)
6	2 (0.9)	1 (0.4)	2 (0.9)	0 (0.0)	5 (0.6)

Data are expressed as n (%).

TC, total cholesterol; CV, coefficient of variation; PCI, percutaneous coronary intervention
